# Insights into the Mechanism for Vertical Graphene Growth by Plasma-Enhanced Chemical Vapor Deposition

**DOI:** 10.1021/acsami.1c21640

**Published:** 2022-01-10

**Authors:** Jie Sun, Tanupong Rattanasawatesun, Penghao Tang, Zhaoxia Bi, Santosh Pandit, Lisa Lam, Caroline Wasén, Malin Erlandsson, Maria Bokarewa, Jichen Dong, Feng Ding, Fangzhu Xiong, Ivan Mijakovic

**Affiliations:** † National and Local United Engineering Laboratory of Flat Panel Display Technology, College of Physics and Information Engineering, Fuzhou University, and Fujian Science & Technology Innovation Laboratory for Optoelectronic Information of China, Fuzhou 350116, China; ‡ Department of Microtechnology and Nanoscience, Chalmers University of Technology, Göteborg 41296, Sweden; § Key Laboratory of Optoelectronics Technology, College of Microelectronics, Beijing University of Technology, Beijing 100124, China; ∥ Division of Solid State Physics and NanoLund, Department of Physics, Lund University, Box 118, S-22100 Lund, Sweden; ⊥ Department of Biology and Biological Engineering, Chalmers University of Technology, Göteborg 41296, Sweden; # Department of Rheumatology and Inflammation Research, University of Gothenburg, Göteborg 41346, Sweden; ¶ Centre for Multidimensional Carbon Materials, Institute for Basic Science, Ulsan National Institute of Science and Technology, Ulsan 44919, Korea; ∇ The Novo Nordisk Foundation Center for Biosustainability, Technical University of Denmark, 2800 Kgs. Lyngby, Denmark

**Keywords:** vertical graphene, plasma-enhanced chemical vapor deposition, GaN nanowires, nanoparticles, 2D materials

## Abstract

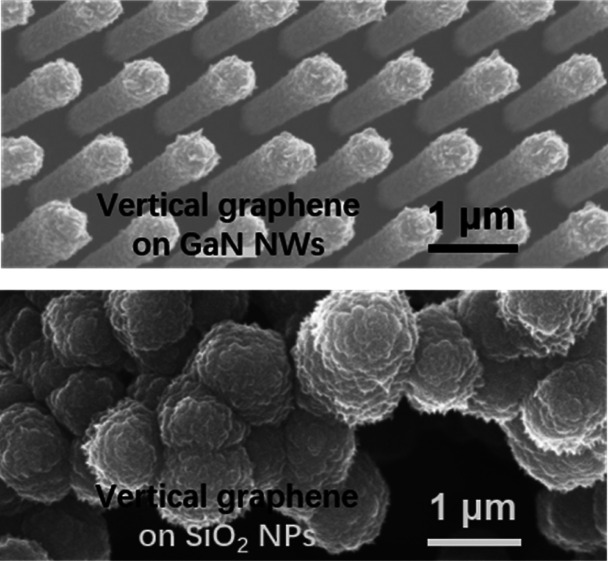

Vertically oriented graphene (VG) has attracted attention for years, but the growth mechanism is still not fully revealed. The electric field may play a role, but the direct evidence and exactly what role it plays remains unclear. Here, we conduct a systematic study and find that in plasma-enhanced chemical vapor deposition, the VG growth preferably occurs at spots where the local field is stronger, for example, at GaN nanowire tips. On almost round-shaped nanoparticles, instead of being perpendicular to the substrate, the VG grows along the field direction, that is, perpendicular to the particles’ local surfaces. Even more convincingly, the sheath field is screened to different degrees, and a direct correlation between the field strength and the VG growth is observed. Numerical calculation suggests that during the growth, the field helps accumulate charges on graphene, which eventually changes the cohesive graphene layers into separate three-dimensional VG flakes. Furthermore, the field helps attract charged precursors to places sticking out from the substrate and makes them even sharper and turn into VG. Finally, we demonstrate that the VG-covered nanoparticles are benign to human blood leukocytes and could be considered for drug delivery. Our research may serve as a starting point for further vertical two-dimensional material growth mechanism studies.

## 1 Introduction

Vertically oriented graphene (VG) or so called carbon nanowalls are three-dimensional (3D), self-supported, interconnected networks of free-standing graphene sheets, whose orientations are approximately perpendicular to the substrate surface. Besides the fact that it possesses extraordinary properties of ordinary graphene, VG itself also has unique properties of high specific surface area, mechanical stability, open reactive graphene edges, easy functionalization, and special optical, thermal, and electrical properties.^1−7^ Due to these properties, VG has a high potential in a wide range of applications. It has caught a lot of interest in various sectors such as field emission, gas- and bio-sensors, blackbody coating, spintronics, and so forth.^8−14^

Although it has been reported that VG can be grown by radio frequency sputtering,^15^ plasma-enhanced chemical vapor deposition (PECVD) is by far the dominant technique to achieve the VG synthesis.^11^ PECVD provides several advantages compared to other techniques, including a lower substrate temperature, a higher growth rate, and a better control on nanostructure ordering due to the presence of energetic electrons, excited molecules/atoms, free radicals, photon, and other active species in the plasma region. Compared with thermal CVD which is based on neutral gas chemistry, PECVD is a more complex process, which can manipulate the morphology and structure of VG by altering the plasma source and adjusting a series of parameters.^16−18^

The growth mechanism of VG by PECVD has been studied and explained by many groups, but it still has some questions left to be answered. There are many factors whose roles have not been clearly revealed, such as the internal stress building up during the growth, the feature of anisotropic growth of VG, and the local electric field introduced by the plasma sheath in PECVD.^1^ Some authors think that the growth starts from the mismatch of the graphitic layer in the “carbon onion” and/or buffer layer, which serves as the nucleation center.^11^ Then, the VG continues its growth by surface diffusion after the deposition of carbonic atoms which are dissociated from the precursor gas in the plasma. Meanwhile, the surface of VG can also be etched with the atomic hydrogen in the growth chamber.^19^ Zhao et al. have developed a continuum model describing the mentioned mechanism and explaining the convergence tendency at the edges of the VG sheets.^20^ On the other hand, there are also evidences implicating that the electric field may be important in promoting the growth of VG.^21^ Controlling the coverage of the electric field can be used as a fabrication technique for patterning the VG in nanodevices.^22^ However, these studies are generally not conclusive. For example, in ref (22), it is observed that VG prefers to grow on Au rather than on SiO_2_ surfaces, and it is explained by the presence of much stronger electric field in the vicinity of Au as compared with SiO_2_. Nevertheless, it could also be possibly explained by a much better catalysis effect on the gold surface compared with SiO_2_. Therefore, up till now, to our knowledge, there is no convincing and direct evidence showing that electric field does promote the growth of VG and explaining its mechanism in detail. We do believe that the central role in the growth mechanism of VG is played by the electric field. We think that the carbonic ions diffusing along the surface of VG are pulled to the direction of the relatively stronger electric field in the structure, thus resulting in the denser and longer VG growth as compared to the other areas where the fields are weak.

In this work, we will demonstrate more and much stronger supporting evidences confirming the effect of local electric field in enhancing the growth of VG on flat substrates and on nanostructures. VG has been first deposited by PECVD on GaN sharp-tipped nanowires to reveal the relation between the effect of local electric field and the VG growth. We have proposed a model to explain the strong-electric field-enhanced VG growth on sharp-tipped GaN nanowires from a cold-walled PECVD chamber. Another experiment is set up to observe the screening effect of local electric field using stainless-steel meshes that can partly block the electric field formed in the plasma sheath while operating the PECVD. The field on the substrate surface is screened to different extents, and the growth results which are studied by using scanning electron microscopy (SEM) are compared. The positive effect of the field on VG growth is again confirmed without ambiguity. We have also succeeded in fabricating a novel material, which is VG-coated silica nanoparticles. Previously, there was an attempt to wrap nanoparticles with graphene oxide^23^ for drug delivery application. However, to our knowledge, there has been no report on wrapping nanoparticles with VG. It is found that the VG grows perpendicular to the SiO_2_ nanoparticle surfaces instead of growing perpendicular to the substrate, which indicates that the VG growth direction is in line with the local field. We have experimentally found that the VG growth actually starts from flat graphene growth. By virtue of the electric field of the plasma, charges accumulate on the graphene flakes. Numerical calculation is carried out, and it is found that due to the Coulombic repulsive force, the flakes change their growth direction and stick out vertically. Also, because sharper features on the substrate are places where the field is stronger, they attract more active species that promote the growth, which makes the features even sharper. This positive feedback loop further boosts the VG growth. Thus, the theoretical results explain the experimental findings well. Finally, for applications, we have cultured human peripheral blood mononuclear cells (PBMCs) on top of our substrates decorated with the VG-coated nanoparticles on their surfaces. The biological interaction between the cells and the VG-coated particles is observed. Despite the observed interaction with VG, we found no harmful consequences to the cells with respect to viability and cytokine production, which indicates its potential application in drug delivery. Our results can be seen as a systematic investigation of the electric field influence on the VG growth with a specific value to material growth scientists and engineers working in the field and can be extended to the vertical growth of two-dimensional (2D) materials beyond graphene.

## 2 Results and Discussion

### 2.1 VG Growth on GaN Nanowires

**Figure 1:**
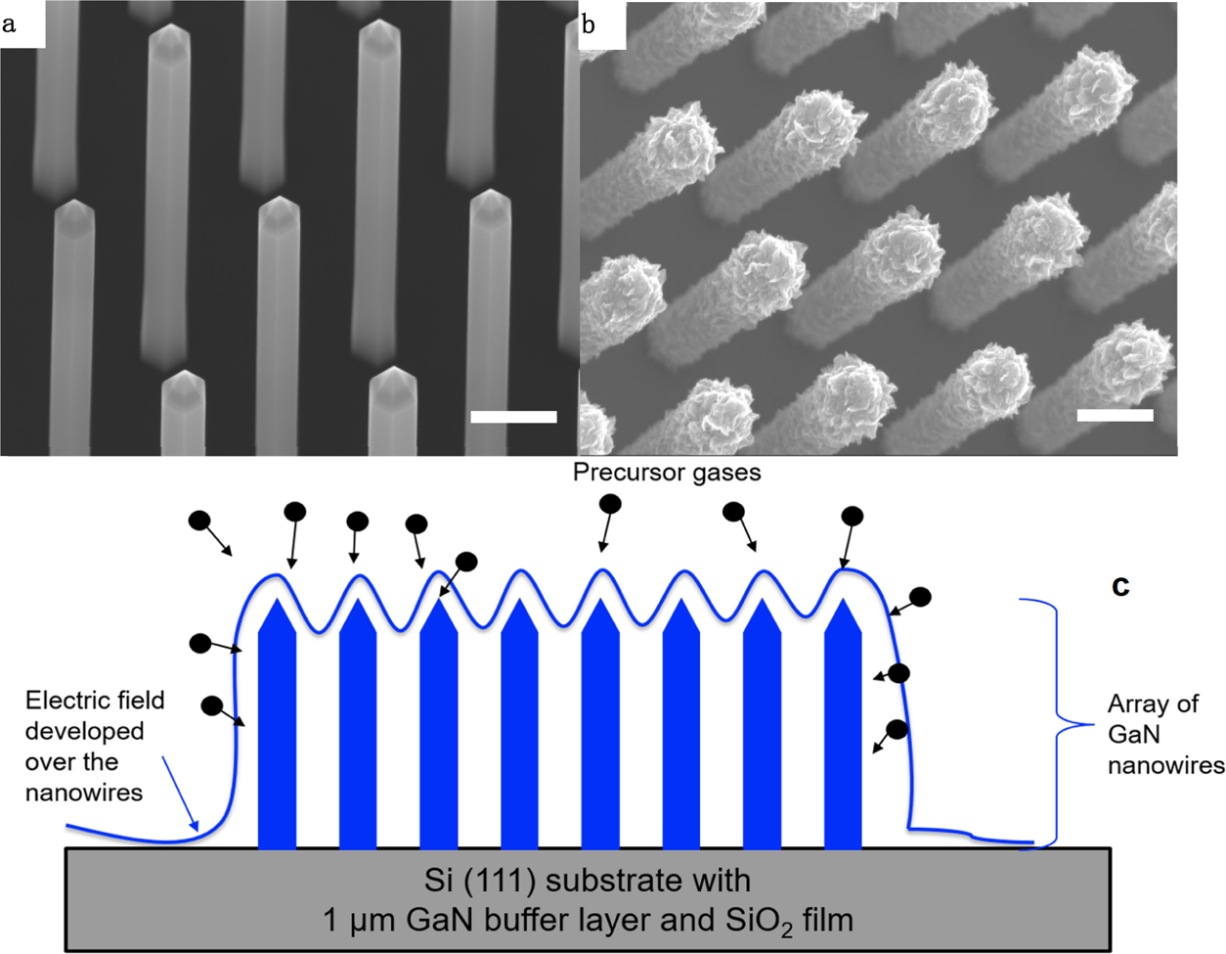
SEM images of GaN nanowires (a) before and (b) after the VG growth. In (b), it is seen that the VG coating on the GaN nanowires has a dominant effect of the local electric field, making the VG denser and longer in the tip region of the nanowires than on its body. Scale bar: 0.4 (a) and 0.5 μm (b). The schematic illustration of the strong local electric field around the sharp tips and its enhancement effect on the VG growth is shown in (c).

In corona discharge theory, charges tend to accumulate toward sharp edges, and electric field is the strongest where the radius of curvature is small. Therefore, trying to grow VG on sharp surfaces is the best way to clarify whether the local electric field is indeed a factor for promoting the VG growth or not. Thus, in order to study the effect of electric field and fully understand its growth mechanism, we grow arrays of GaN nanowires on GaN/Si substrates from nanoscale openings patterned in a SiN_*x*_ mask by electron beam lithography, as shown in Figure 1a. The nanowires grow selectively from the SiN_*x*_-free openings, and the nanowires have a height of about 1.5 μm and a width of about 200 nm. After standard organic cleaning, this nanowire pattern is used for the VG growth. The VG is then deposited in our cold-walled PECVD chamber. A typical Raman spectrum is shown in Figure S1, which is taken from a VG sample grown for 10 min with our standard parameters as reported elsewhere.^24^ Based on the G, 2D, and D Raman peaks, it can be concluded that the grown VG is sp^2^-hybridized. Figure 1b shows SEM images after the growth of VG. It can be seen that the VG grows all over the wires. From Figure S2, it is clear that the VG also grows on the SiN_*x*_ mask between the nanowires on the substrate. However, the VG flakes at the tips of the nanowires are much denser and longer than those grown on the nanowire bodies (see Figure 1b). The VG flakes at the tips are measured to be about 100–150 nm in length. In Figure S2, the flow rate of the C_2_H_2_ precursor gas and the time used in the growth is modulated. It shows that when the flow rate and growth time are high enough, the effect of electric field on the VG growth can be observed distinctly.

The observed phenomenon can be well explained by the enhanced electric field around the tips. When the nanowire structure is covered with the plasma sheath in the PECVD machine, it will develop a local electric field profile based on its geometry. For our highly crystalline GaN nanowires, because of their hexagonal pyramid-shaped apexes as shown in Figure 1a, they naturally hold the strongest electric field at their sharp tips, which can result in attracting carbon ions in the plasma for adsorption and diffusion there, leading to an enhanced VG growth mode. It was, thus, expected to have the densest and also the longest VG growth at these pointing tips. We note that because this type of semiconductor nanowires is highly crystalline and forms highly ordered arrays, it is probably the best candidate to study the electric field effect. To the best of our knowledge, this is the first experiment of growing VG on GaN semiconductor nanowires. VG flakes have been grown on carbon nanotubes as well.^25^ However, because today’s technology for producing carbon nanotubes is not as neat as that for semiconductor nanowires and, most importantly, carbon nanotubes generally have round ends,^26^ the results of the electric field effect on the VG growth there are not very conclusive.

### 2.2 Electric Field Screening Effect with Stainless-Steel Meshes

**Figure 2:**
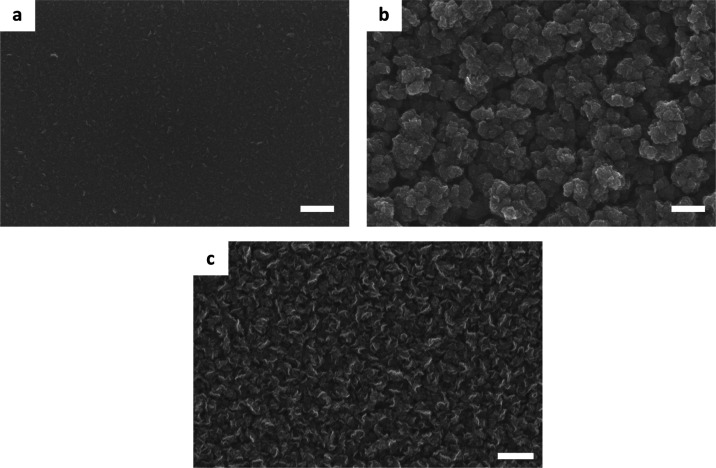
SEM images of the VG grown on the 2 in. SiO_2_/Si substrate surface when the electric field is screened using stainless-steel meshes to different degrees. (a) Typical VG growth in region I in Figure S3, where the electric field is almost totally screened using a small mesh with the hole size of about 140 μm. An almost entire inhibition of VG growth is observed. (b) Electric field is partly blocked using a metal mesh with the hole size of about 400 μm, corresponding to region II in Figure S3. There is 3D growth, but with no sharp edges and also the “VG” is grown as clusters rather than “free-standing” sheets. (c) In region III where there is no screening, the VG growth is back to normal and flower-like vertical flakes are observed as expected. Scale Bar: 0.5 μm for all three images.

In our experiment, we have found that if remote plasma instead of local plasma is used in the PECVD, the VG can be hardly grown. This is an indication that in order to initiate the VG growth, the samples have to be immersed in the plasma sheath, where the electric field is the strongest. In the following, we design another experiment to investigate deeper on the effect of electric field influencing the growth. In the setup, two stainless-steel meshes with different hole sizes are used to screen part of the electric field. In this experiment, the plasma is local where its electrode is located directly below the graphitic heater plate. The substrate, which is a 2 in. silicon wafer with 400 nm SiO_2_ coating, is placed on the heater. The plasma electrode is powered with positive voltage while the heater is grounded. The huge voltage difference is then used to ignite the plasma, and the electric field from the plasma sheath is formed from slightly above the heater. The two stainless-steel meshes are placed less than 1 mm above the heater and cover some part of the substrate, as shown in Figure S3. The sample area can be divided into three regions (see Figure S3c). Region I is covered with mesh 1 with small holes (140 μm diameter), region II is covered with mesh 2 with slightly bigger holes (400 μm diameter), and region III is the uncovered area. Due to the discharge effect, where the sparkles are vividly seen in Figure S3b, some areas have not been effectively screened, which means that at some spots, electric fields are leaked to the sample beneath the meshes. Notwithstanding this, there do exist some areas that have been successfully screened out of any electric field, as outlined by the dashed lines in Figure S3c. The SEM images of the VG after the PECVD growth are summarized in Figure 2, where Figure 2a is taken from region I. It is the area where most of the electric field is screened out using a metal mesh with small holes. The growth of VG is almost totally inhibited by the absence of the electric field influence. Because the mesh is only screening out the electric field, the precursor gas can still reach the substrate surface, which leads to the growth of ordinary few-layered flat graphene. Although for the area in Figure 2b, the electric field is also blocked but with somewhat more leakage due to the bigger size of the mesh holes, the image shows that there is some 3D growth of VG-like structures. However, they are missing the feature of sharp edges, and they also grow as clusters instead of “free-standing” vertical sheets. Both growth results demonstrate the importance of local electric field that is heavily influencing the growth of VG. On the SiO_2_/Si substrate where it is not covered with any steel mesh, the typical SEM image is represented in Figure 2c. Not surprisingly, the VG in this area shows a network of standing graphene flakes with the feature of sharp edges, which is expected for a standard VG growth.

### 2.3 VG Growth on Nanoparticles

**Figure 3:**
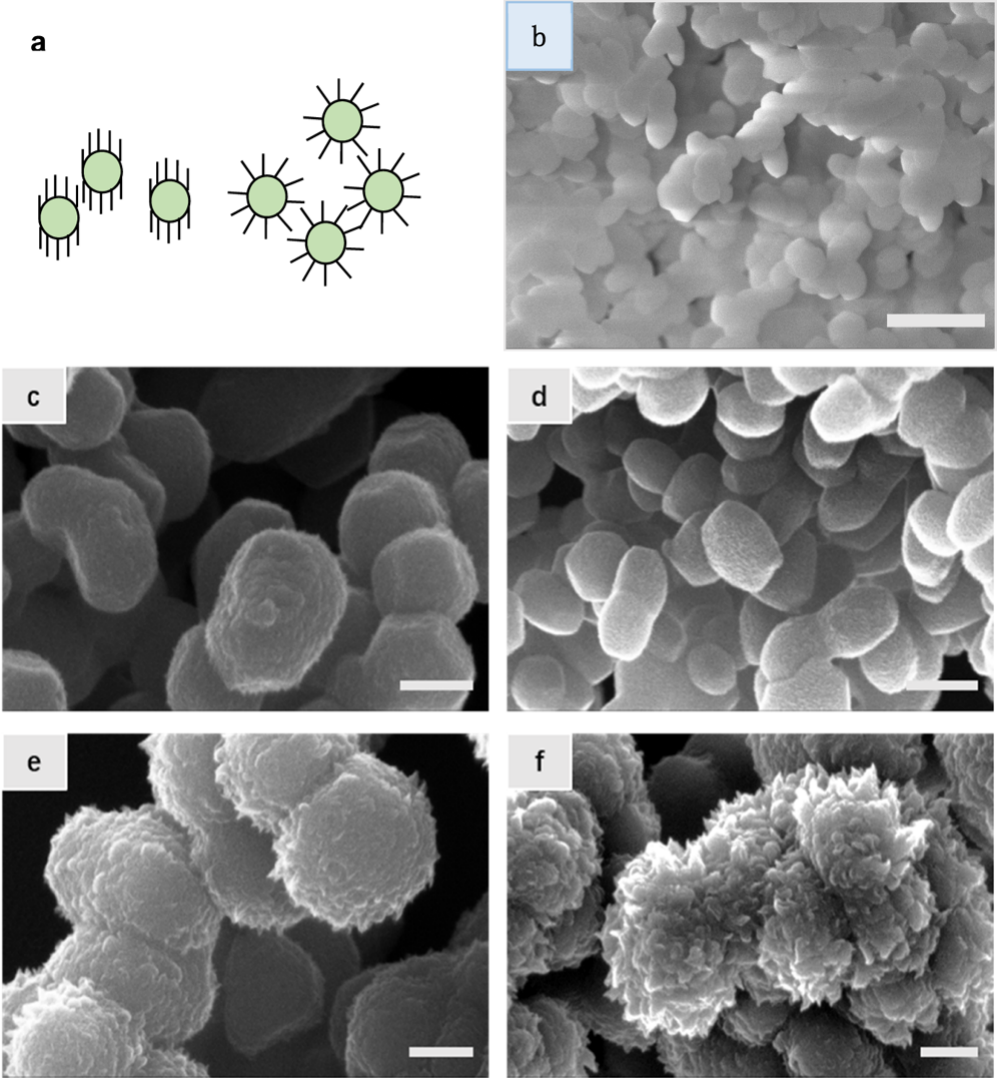
(a) Schematic illustration of two possible growth modes of the VG on nanoparticles. On the left, the flakes are perpendicular to the substrate. On the right, the flakes are alongside the normal directions of the nanoparticles, that is, the directions of the local field. The latter growth mode is confirmed by the experiments. (b) SEM image of the SiO_2_ nanoparticles that are drop-coated on the substrate surface. The particles are about 450–500 nm in size. (c–f) SEM images of the VG grown on the silica nanoparticles with different flow rates of C_2_H_2_ and growth times. The flow rates and growth times are: (c) 5 sccm and 3 min, (d) 5 sccm and 5 min, (e) 15 sccm and 3 min, and (f) 15 sccm and 5 min. Scale bar: (b) 2 μm, (c) 500 nm, (d) 1 μm, (e) 400 nm, and (f) 500 nm.

Another experiment we will carry out is to grow VG on nanoparticles. To our knowledge, this has not been carried out before. One of the motivations for this experiment is to further study the local electric field effect. When the VG is grown on the nanoparticles, it could take two possible orientations as schematically drawn in Figure 3a. On the left part of this figure, the VGs are pointing upward, which at first glance seems to be logical. However, because the electric field in the plasma follows the shape of objects that are immersed in the sheath, the local field direction should be perpendicular to the surfaces of the particles. Hence, the profile on the right part of Figure 3a is expected. Described below is our actual verification of this hypothesis.

SiO_2_ nanoparticles dispersed in isopropanol are drop-coated on the SiO_2_/Si substrate and left for drying overnight. These commercial nanoparticles have an approximately round or hexagonal shape with a typical size of 0.45–0.5 μm and are self-organized on the substrate as shown in Figure 3b. We then grow VG on the sample by the same PECVD method. With the flow rate and growth time being carefully tuned, the VG height can be well controlled. Figure 3c–f shows the SEM images of the VG-coated SiO_2_ nanoparticles with different VG heights. The growth parameters are written in the figure caption. As expected, higher flow rates and longer deposition time lead to longer VG flakes. Importantly, it also shows that the growth of the VG on the nanoparticles (see Figure 3e,f) is in the normal direction with respect to the surfaces of the particles or, in other words, in the same direction of the electric field formed on their surfaces. We have also grown VG on Fe_3_O_4_ nanoparticles (∼10 nm in size) in the same manner, and similar results are observed.

### 2.4 Deeper Look into the Electric Field-Assisted Growth Mechanism of VG

**Figure 4:**
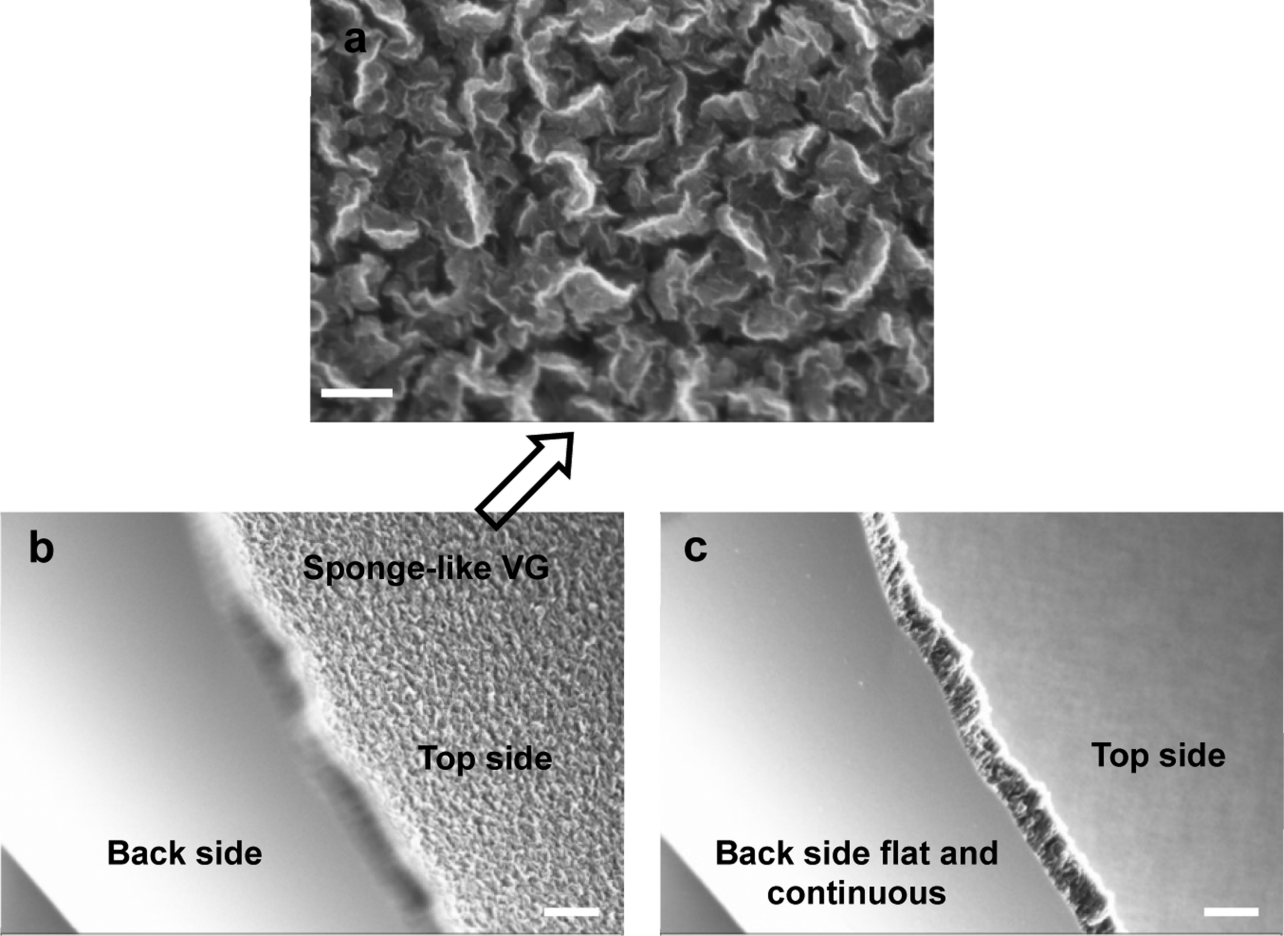
(a) SEM micrograph of a VG film grown by PECVD on the SiO_2_/Si substrate. The flakes are rooted in the substrate and stick out vertically in 3D space. (b,c) SEM images with focus on the top (b) and back (c) side. They show that the porous VG film is mechanically rather strong and can be peeled off from the substrate. On the top side, numerous covalently bonded graphene flakes are seen, whereas the backside is totally flat and continuous. Most importantly, it directly proves that this type of graphene first grows horizontally, and when the thickness slightly increases, the growth mode changes to the vertical growth. The SEM micrographs in (b,c) are taken from an angle. Scale bar: 200 nm in (a) and 1 μm in (b,c).

Based on the experimental results we have got, we are able to dig deeper into the growth mechanism of VG. It is known that when the VG grows, it usually starts with horizontal graphene growth,^27^ which can be regarded as a buffer layer. This is a logical and natural scenario. However, there has not been very clear and direct observation of such a horizontal layer in the literature. Here, we present a concrete evidence of the horizontal graphene underneath the VG during the initial growth stage. Figure 4 shows the SEM image of a VG film grown on a 300 nm SiO_2_/Si flat substrate using our standard recipe. Figure 4b shows the SEM image (top view) of the VG, where the graphene flakes are tightly connected to each other, most likely covalently bonded, forming a porous film. Therefore, the VG film is mechanically rather strong. It is known that in a liquid environment, the surface tension pulls toward any solid that the liquid is in contact with. For our VG, however, we have found that even if it is directly taken out of water and blow-dried with N_2_, the graphene will not collapse. It even keeps its integrity when it is mechanically delaminated from the substrate (see Figure 4b, where both the top and back sides are visible). When the focus is set to the topside, the typical VG morphology is observed, as shown in the enlarged micrograph of Figure 4a. When focusing on the backside (Figure 4c), it is seen to be super flat, meaning that all the vertical flakes are rooted in a horizontal graphene film. Observing the cross-section, the film thickness is on the order of few hundred nm. Figure 4 points out an important fact that the deposition indeed starts from a horizontal growth mode.

**Figure 5:**
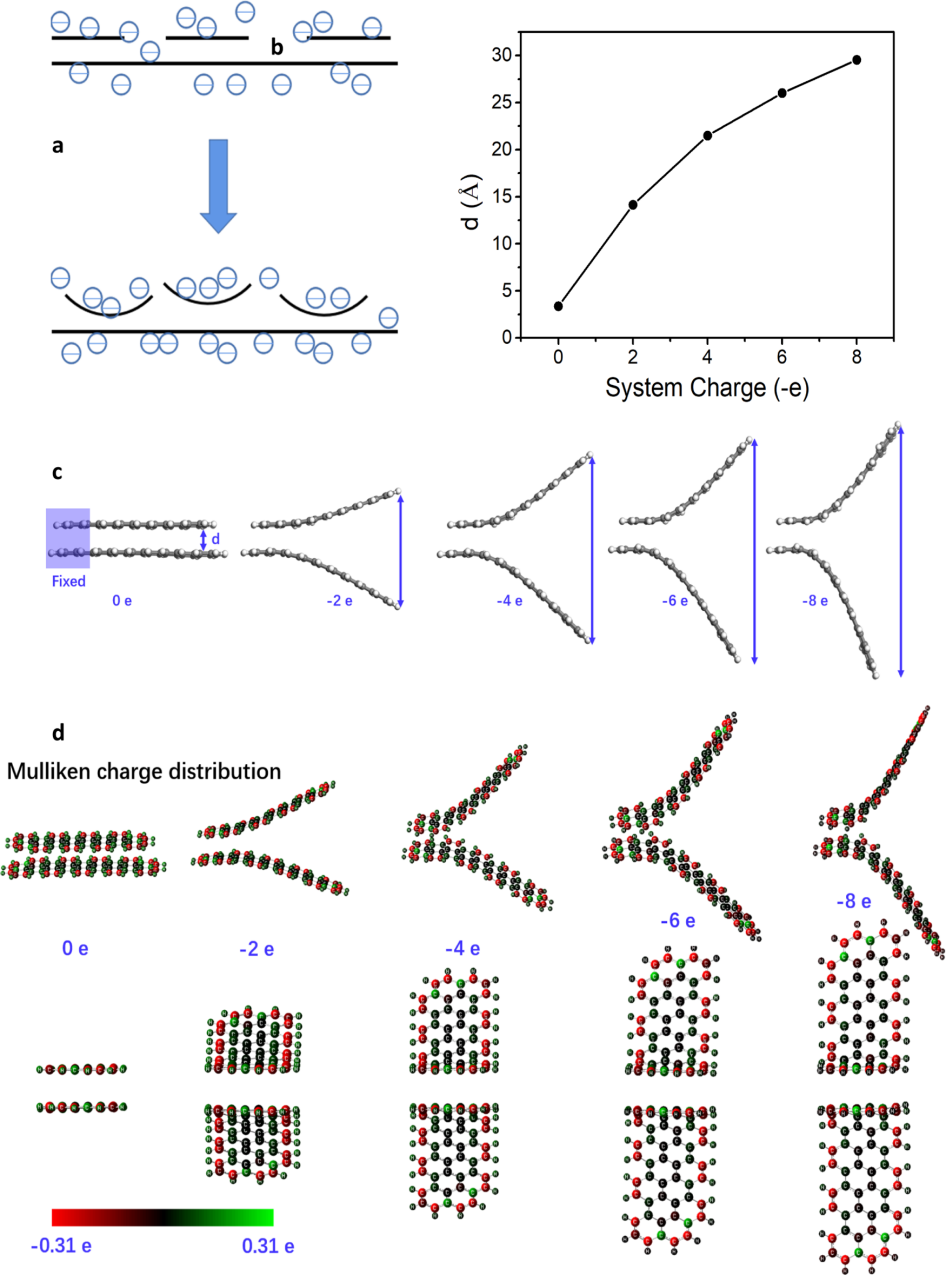
(a) Schematic demonstration of the change from 2D to 3D growth due to the charge accumulation. When the charge density is high enough, the Coulombic force would overcome the van der Waals interaction in multilayer graphene, leading to the formation of VG. (b–d) Simulation results. (b) Distance between the two graphene tips plotted as a function of the system charging value. (c) Same data, showing that with the increase in the system charging value, the distance between the tips increases significantly due to the electrostatic repulsive force between the tips. (d) Simulated Mulliken charge distribution. With the increase in the system charging value, the Mulliken charges (added electrons) tend to distribute near the circumference.

In plasma, electrons moves much faster than positively charged ions. Therefore, what usually happens is that electrons will transfer to the substrate and charge it negatively. An electric field is thus produced at the interface between the substrate and the plasma at equilibrium. This layer is called the Debye sheath, and the thickness of such a layer is called Debye length. Because the VG growth starts with horizontal graphene deposition, in plasma, these graphene layers are negatively charged in the sheath, as schematically shown in Figure 5a. We propose that the most important reason for the growth mode to change from 2D to 3D is the charge accumulation. By virtue of the Coulombic repulsive force, the graphene layers are split and start to point upward. We have carried out a simulation on this growth mechanism. Figure 5b shows the split distance between the graphene layers as a function of the charge. Eight charges are enough to separate the two neighboring graphene layers by ∼3 nm (Figure 5b,c). Clearly, the morphology of the originally horizontal multilayer graphene is heavily influenced by its charge status. Figure 5d shows the Mulliken charge distribution on the graphene flakes. It is found that with the increase in the system charging value, the added electrons tend to distribute around the circumference.

We also propose another important VG growth mechanism that is based on the local electric field as well. When the 2D to 3D growth transition happens as described above, some places start to appear slightly higher than others. Therefore, the local field is stronger at these higher points because of the smaller radius of curvature. The decomposed precursor consists of charged ions, which are hence directed toward these positions by the electric field. As a result, the VG grows more in these areas as compared to other places. Little by little, they become even sharper and protruded, which in turn makes the field even stronger, forming a positive feedback loop. The two growth mechanisms are both electric field-initiated, and they work simultaneously in the growth chamber, facilitating the VG deposition. This explains why when the electric field is screened out, the VG growth literally ceases. Also, if remote PECVD is used instead as the samples are not immersed in the plasma sheath where the electric field is strong, although the precursors can still be effectively cracked, the VG growth is not feasible any more.

### 2.5 Interaction of VG-Covered Nanoparticles with Human Cells for Potential Drug Delivery Applications

It is proposed that graphene has a high potential in drug delivery.^23,28^ However, up till now, there are still important issues remaining unclarified. For example, whether graphene is harmful to human cells remains arguable. Nanoparticles are a type of traditional materials used in drug delivery. VG flakes have a high surface-to-volume ratio and can be grown virtually on any stable material that withstands high temperature. Therefore, we propose to combine nanoparticles with VG for drug delivery applications. Due to the local electric field-based growth mechanism, the VG flakes stick out toward every direction (see Figure 3e,f), which is in favor of drug delivery. The high specific area of VG may increase SiO_2_ particles’ functionality. In the following, we demonstrate that they are indeed human cell-friendly.

**Figure 6:**
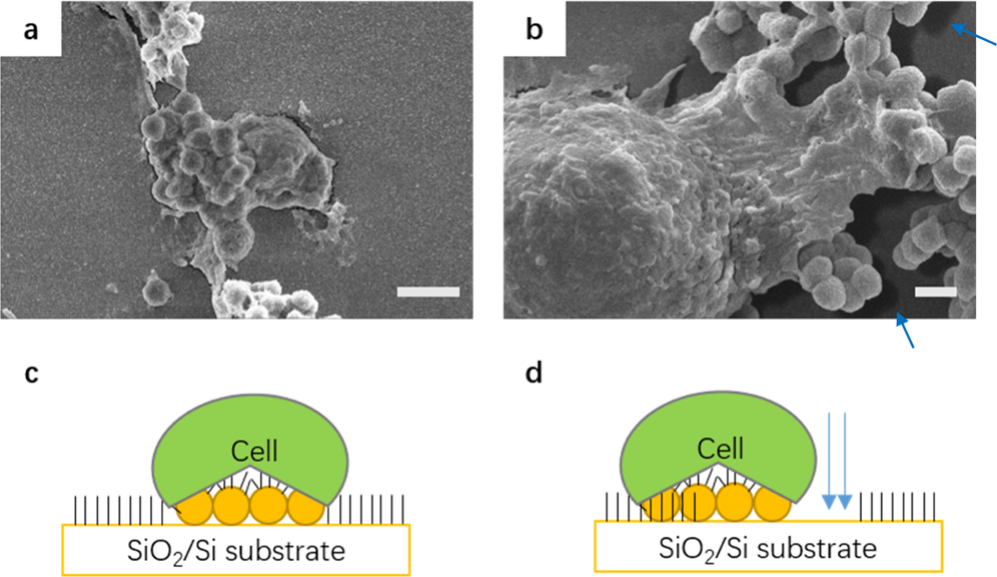
(a) SEM image showing that a cell interacts with a cluster of VG-covered nanoparticles either by covering or internalizing the nanoparticles. The shape of the nanoparticle cluster may be seen under the cell membrane. (b) Cell is interacting with a cluster of nanoparticles. The cell forms sprouts which are reaching out for the nanoparticle clusters. (c) Schematic illustration of the PBMC covering or internalizing nanoparticles on the SiO_2_ substrate. (d) Schematic illustration of the PBMC movement after the internalizing of nanoparticles. The arrows point at the area where the nanoparticles have been moved by the cell, which leaves dark borders in the SEM image in (b). Scale bar: 3 (a) and 1 μm (b).

We first coat SiO_2_ nanoparticles with height-controllable VG. PBMCs are cultured on the SiO_2_/Si substrate that is deposited with VG-coated SiO_2_ nanoparticles. We find that the VG, even with its sharp edges, does not endanger the PBMCs. Figure 6 shows the interaction between the cells and the VG-coated SiO_2_ particles. The cell morphology is clearly visible and preserved. The cells interact and engulf the particles presenting normal physiological function. In Figure 6a, the cell covers or internalizes a cluster of SiO_2_ nanoparticles. In Figure 6b, the cell forms sprouts, reaches out, grasps, and internalizes a large number of nanoparticles. Interestingly, the cell demonstrates certain ability to change its position even after “eating” the nanoparticles. In Figure 6b, we can see a dark border around the cell. It is because the particles therein have been removed by the cell, and the VG-free surface of the SiO_2_/Si substrate is exposed. This procedure is schematically illustrated in Figure 6c,d. This experiment demonstrates that the PMBCs are still healthy after interacting with the VG-coated SiO_2_ particles. This result expands the potential application of VG in the pharmaceutical field. Some additional SEM images can be found in Figure S4.

## 3 Conclusions

In summary, the mechanism of VG growth in PECVD has been systematically studied. The VG is deposited on GaN nanowires, and it is found that the VG prefers to grow at the sharp tips, where the local electric field is the strongest. Furthermore, the field is particularly screened by using metal meshes with different sizes of holes to verify the vital effect of the field. On SiO_2_ nanoparticles, it is found that the VG always grows perpendicular to the surface of the particles, namely, along the direction of electric field. Theoretical calculation points out that when the VG grows, the electric field can accumulate electrons on the flakes, which eventually turns the growth mode from 2D into 3D by virtue of the Coulombic repulsion. Also, sharper points are places where the electric field is stronger, which will direct the charged precursor species to these points and promote the growth. Finally, we demonstrate a potential application for drug delivery using the VG-covered SiO_2_ particles, whose surface to volume ratio has been maximized. Previously, the understanding of VG growth mechanism had been very vague. Although the electric field is believed to play a role in the VG growth, the direct evidence of this role and exactly how it works has been missing in the literature. Our research unambiguously reveals the electric field-based growth mechanism and can be of a reference value to VG growth designers in the field. For example, although the precursors may be effectively cracked, remote PECVD may not work for the VG growth because the samples are placed outside of the intensive electric field area. Finally, we point out that our results may be useful as a guideline for exploring a family of vertically aligned 2D materials other than graphene. A typical example is h-BN, which is a 2D insulator. The growth mechanism is still under investigation,^30^ but vertical h-BN flakes have been realized by PECVD already.^31^ Vertical 2D semiconductors, for example, MoS_2_ is also synthesized, although with methods other than PECVD.^32,33^

## 4 Experimental Section

### 4.1 PECVD Growth of VG

VG was grown on the substrate in a cold-walled low-pressure PECVD reactor (Black Magic, Aixtron). The substrate was heated to 775 °C and annealed for 1 min with the mixing of 20 sccm H_2_ gas and 1000 sccm Ar gas. The plasma is then turned on under a DC bias with the power of 75 W. The plasma voltage is 800 V with the current limit of 0.5 A. The actual growth was initiated by introducing C_2_H_2_ (usually 15 sccm) and maintained typically for 10 min. After the growth, the system was evacuated to <0.2 mbar and cooled down.

### 4.2 Preparation of GaN Nanowires and SiO_2_ Nanoparticles

Pure GaN nanowires with no indium or aluminum compositions were grown by metal organic CVD on (0001)-oriented GaN/Si substrates [the Si is (111)-oriented] with a 30 nm-thick SiN_*x*_ mask. Electron beam lithography was used to fabricate openings in the SiN_*x*_ mask, where the GaN nanowires grew.

The SiO_2_ nanoparticles were actually Zr-SBA-15, a kind of mesoporous SiO_2_ nanoparticles often used in drug delivery. The powders were uniformly dispersed in isopropanol or acetone with ultrasonic treatment. Two or three drops of the solution were then drop-coated on the 1 × 1 cm^2^ SiO_2_/Si substrate and left overnight for drying.

### 4.3 Cell Culture

Human PBMCs were isolated from venous peripheral blood of healthy volunteers using density gradient separation on Lymphoprep (Axis-Shield PoC As, Norway). Cells were washed and resuspended in Iscove’s medium (containing 1% l-glutamine, 5 × 10^–5^ M β-mercaptoethanol, 50 μg/mL of gentamycin sulfate, and 10% fetal calf serum) at 2 × 10^6^/mL and set on the slides coated with VG-coated SiO_2_ nanoparticles. Cell cultures were stimulated with LPS (50 ng/mL) for 24 and 72 h in a humidified atmosphere containing 5% CO_2_ at 37 °C. The PBMCs were fixated on the substrates. The samples were then coated with a 5 nm gold layer by sputtering before the SEM observation.

## 5 Theoretical Calculation Details

All calculations were carried out by using the Gaussian09 package with the B3LYP hybrid functional method and 6-31G(d) basis.^29^ As shown in Figures 5 and S5, two parallelly stacked graphene nanoribbon flakes were constructed to represent few-layered graphene in experiments. Different amounts of charges were added to the calculation model to investigate the charge effect on the structure of few-layered graphene. The structure was optimized with its one end fixed and the forces of all the other atoms relaxed to less than 2 × 10^–4^ Hartree/Bohr.

## Supporting Information Available

Typical Raman spectrum of the VG film confirming the graphitic structure, additional SEM images of the VG gron on GaN nanowires with different flows and times, photographs of the experimental setup for screening the electric field using stainless-steel meshes, additional SEM images showing the intimate interaction of the cells and the VG-coated particles, and atomic structures of the theoretical calculation model (PDF)

## Notes

No data sets were generated or analyzed in the current study.
