# Supplementary material for: Insights into the Mechanism for Vertical Graphene Growth by Plasma-Enhanced Chemical Vapor Deposition
Source: ACS Appl Mater Interfaces. 2022 Jan 10;14(5):7152–60. doi: 10.1021/acsami.1c21640 (PMC8832395; doi:10.1021/acsami.1c21640)
Supplement: Supplementary file 1 — am1c21640_si_001.pdf [file am1c21640_si_001.pdf]

Supporting Information

Insights into the Mechanism for Vertical Graphene Growth by Plasma Enhanced Chemical Vapor Deposition

Jie Sun[1,2,a,*], Tanupong Rattanasawatesun[2], Penghao Tang[3], Zhaoxia Bi[4], Santosh Pandit[5], Lisa Lam[6], Caroline Wasén[6], Malin Erlandsson[6], Maria Bokarewa[6], Jichen Dong[7], Feng Ding[7,b,*], Fangzhu Xiong[3], and Ivan Mijakovic[5,8]

[1] National and Local United Engineering Laboratory of Flat Panel Display Technology, College of Physics and Information Engineering, Fuzhou University, and Fujian Science & Technology Innovation Laboratory for Optoelectronic Information of China, Fuzhou 350116, China

[2] Department of Microtechnology and Nanoscience, Chalmers University of Technology, Göteborg 41296, Sweden

[3] Key Laboratory of Optoelectronics Technology, College of Microelectronics, Beijing University of Technology, Beijing 100124, China

[4] Division of Solid State Physics and NanoLund, Department of Physics, Lund University, Box 118, S-22100 Lund, Sweden

[5] Department of Biology and Biological Engineering, Chalmers University of Technology, Göteborg 41296, Sweden

[6] Department of Rheumatology and Inflammation Research, University of Gothenburg, Göteborg 41346, Sweden

[7] Centre for Multidimensional Carbon Materials, Institute for Basic Science, Ulsan National Institute of Science and Technology, Ulsan 44919, Korea

[8] The Novo Nordisk Foundation Center for Biosustainability, Technical University of Denmark, 2800 Kgs. Lyngby, Denmark

[a] Electronic mail: jie.sun@fzu.edu.cn
[b] Electronic mail: f.ding@unist.ac.kr

Figure captions:

Figure S1. Typical Raman spectrum of the VG thin film grown by our standard recipe. 3 distinct bands are found at the following positions: ~1333 cm^{-1} (D band), ~1604 cm^{-1} (G band) and ~2645 cm^{-1} (2D band), which confirms that the material is sp^2 hybridized carbon.

Figure S2. SEM images of the VG growth on GaN nanowires with different flow rates of C_2H_2 (sccm) and growth time (min): (a) 5 sccm, 5 min; (b) 5 sccm, 10 min; (c) 10 sccm, 5 min; (d) 10 sccm, 10 min. Scale bar: 0.5 μm in (a, b) and 0.2 μm in (c, d).

Figure S3. Images of the experimental setup for observing the electric field screening effect by two different sizes of stainless-steel meshes. (a) is the photo taken when the machine is not operating and (b) is when the machine is operating as it can be seen that the substrate is covered by the plasma and by stainless-steel meshes. (c) is the photo of the sample after the VG growth, and the three typical regions (I, II and III) are outlined by dashed lines. I is covered by mesh 1 with small holes, II is covered by mesh 2 with slightly bigger holes, and III is the uncovered area.

Figure S4. Additional SEM images showing the intimate interaction of the cells and the VG-coated SiO_2 nanoparticles. Scale Bar: 3 μm (a) and 10 μm (b).

Figure S5. Atomic structure of the calculation model. This model consists of two graphene nanoribbon flakes, which are stacked with a distance of 3.4 Å. The edges of the graphene flakes are passivated by hydrogen. The carbon atoms in the upper and bottom layers are represented by gray and yellow spheres, respectively. The hydrogen atoms are represented by white spheres.

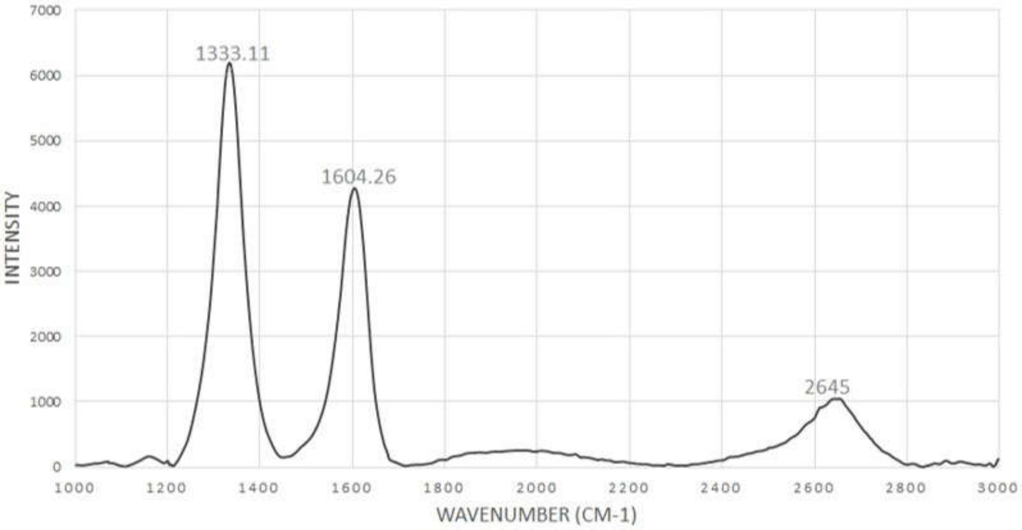

Figure S1

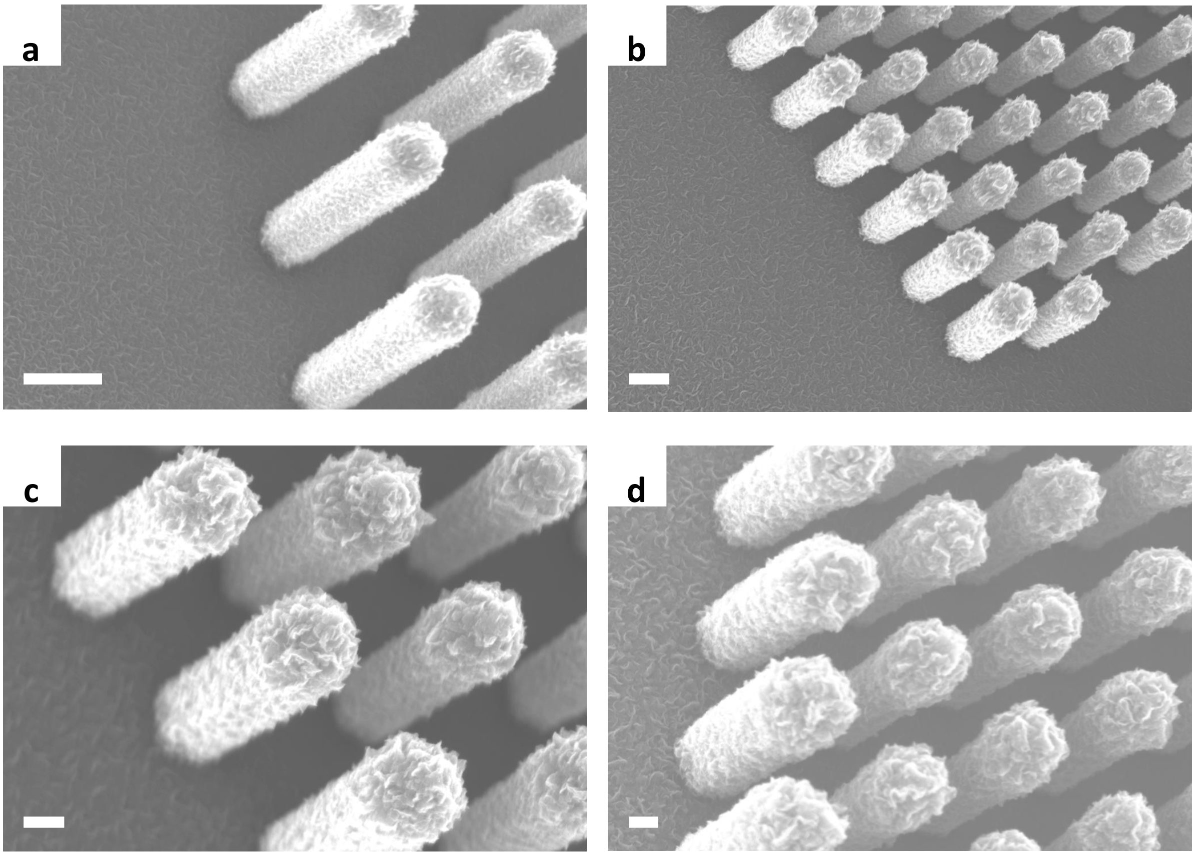

Figure S2

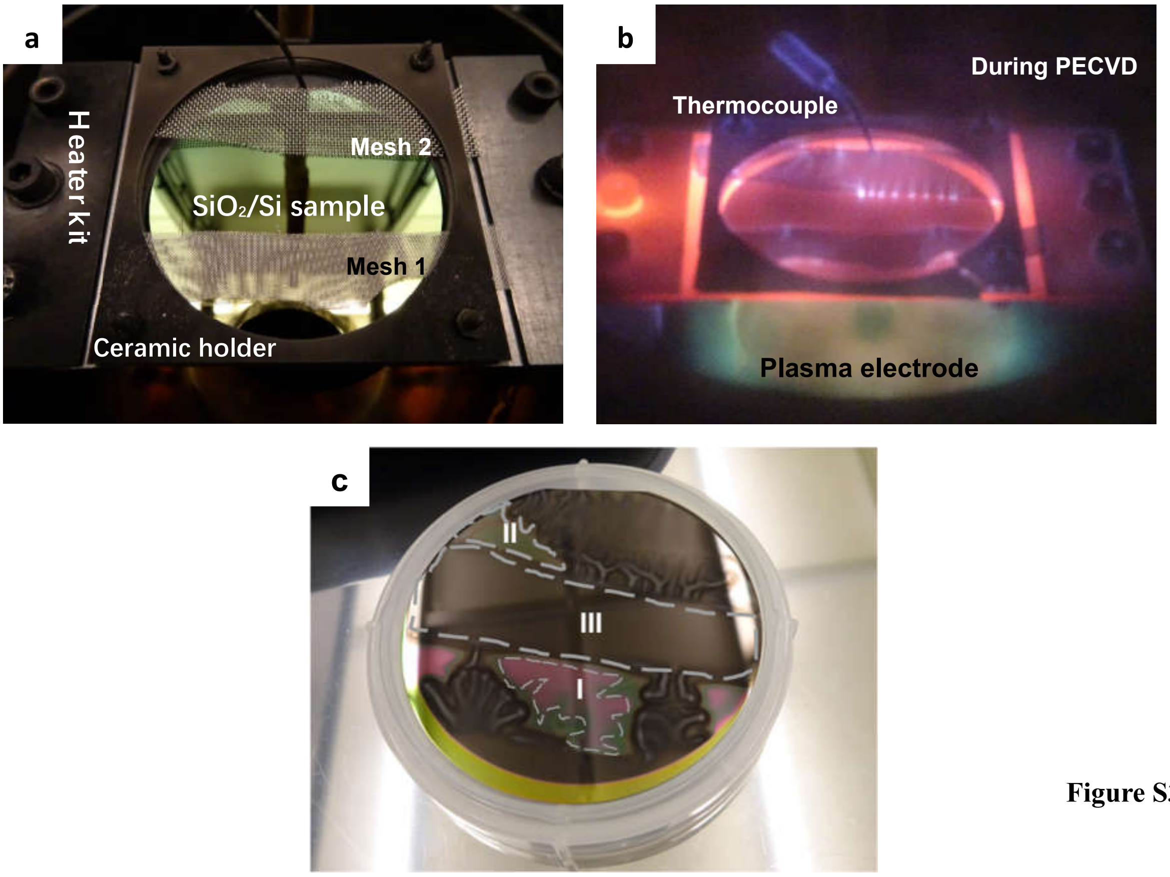

Figure S3

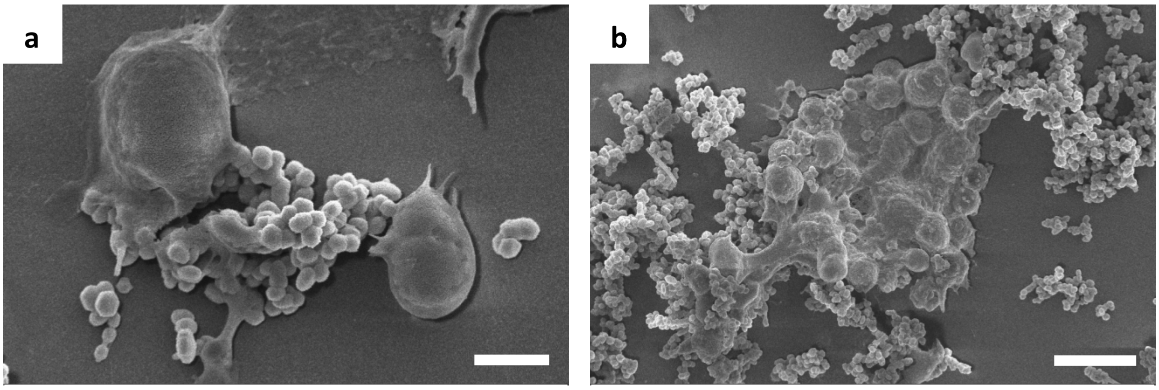

Figure S4

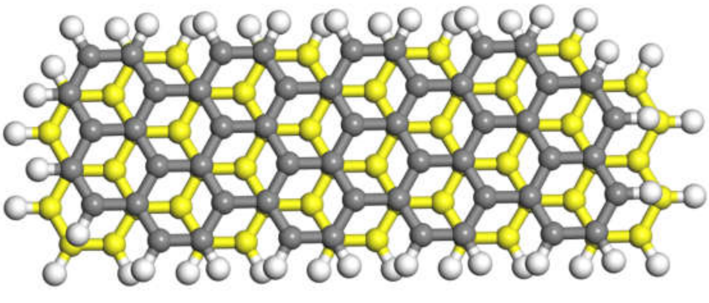

Figure S5
